# Bone Marrow Neutrophils of Multiple Myeloma Patients Exhibit Myeloid-Derived Suppressor Cell Activity

**DOI:** 10.1155/2021/6344344

**Published:** 2021-08-06

**Authors:** Julia Petersson, Sandra Askman, Åsa Pettersson, Stina Wichert, Thomas Hellmark, Åsa C. M. Johansson, Markus Hansson

**Affiliations:** ^1^Department of Laboratory Medicine, Division of Hematology and Transfusion Medicine, Lund University, BMC B13, 22184 Lund, Sweden; ^2^Department of Clinical Sciences Lund, Nephrology, Lund University, Skåne University Hospital, Barngatan 2, 22185 Lund, Sweden; ^3^Department of Hematology, Oncology and Radiation Physics, Skåne University Hospital, 22185 Lund, Sweden; ^4^Region Skåne, Clinical Genetics and Pathology, Skåne University Hospital, BMC C13, 22185 Lund, Sweden; ^5^Sahlgrenska Academy, Institute of Medicine, Department of Internal Medicine and Clinical Nutrition, University of Göteborg, Bruna Stråket 5, Plan 5, 41325 Göteborg, Sweden

## Abstract

Activated normal density granulocytes (NDGs) can suppress T-cell responses in a similar way as myeloid-derived suppressor cells (MDSCs). In this study, we tested the hypothesis that NDGs from blood and bone marrow of multiple myeloma (MM) patients have the ability to suppress T-cells, as MDSC. MM is an incurable plasma cell malignancy of the bone marrow. Like most malignancies, myeloma cells alter its microenvironment to promote tumor growth, including inhibition of the immune system. We found that MM NDG from the bone marrow suppressed proliferation of T-cells, in contrast to healthy donors. The inhibitory effect could not be explained by changed levels of mature or immature NDG in the bone marrow. Moreover, NDG isolated from the blood of both myeloma patients and healthy individuals could inhibit T-cell proliferation and IFN-*γ* production. On the contrary to previous studies, blood NDGs did not have to be preactivated to mediate suppressive effects. Instead, they became activated during coculture, indicating that contact with activated T-cells is important for their ability to regulate T-cells. The inhibitory effect was dependent on the production of reactive oxygen species and could be reverted by the addition of its inhibitor, catalase. Our findings suggest that blood NDGs from MM patients are suppressive, but no more than NDGs from healthy donors. However, only bone marrow NDG from MM patients exhibited MDSC function. This MDSC-like suppression mediated by bone marrow NDG could be important for the growth of malignant plasma cells in MM patients.

## 1. Introduction

Neutrophils are the most abundant white blood cell in the peripheral blood (60-70%) and play an important role in the first line of defense against invading pathogens. They have long been viewed as a homogenous subset with only one function: to seek and kill invading pathogens. However, in recent years, neutrophils have been shown to have a far more diverse role in the immune system, e.g., regulate the adaptive immune response through suppression of T-cell proliferation. The subset responsible for this type of suppression is usually referred to as polymorphonuclear myeloid-derived suppressor cells (PMN-MDSCs) [1]. PMN-MDSCs are increased in patients with different types of cancer and often associated with a poor prognosis [2].

PMN-MDSC and neutrophils are very similar, and density gradient centrifugation is the only way to phenotypically distinguish PMN-MDSC from neutrophils as they end up in the low-density layer [1]. Hence, PMN-MDSCs are sometimes referred to as low-density granulocytes (LDGs), in contrast to normal-density granulocytes (NDGs) [3].

In the last years, NDGs have been shown to also inhibit T-cell responses under certain conditions, for example, when they have been preactivated before coculture with T-cells [4–8]. Aarts et al. have suggested that mature neutrophils, not immature neutrophils, are responsible for the inhibitory effect, and that these cells produce reactive oxygen species (ROS) in order to inhibit proliferation [5].

Multiple myeloma (MM), the second most common hematological malignancy, is clinically characterized by the CRAB criteria—increased calcium levels, renal failure, anemia, and osteolytic bone lesions [9]. In MM, malignant plasma cells proliferate uncontrollably in the bone marrow and produce high amounts of monoclonal antibodies. Many MM patients suffer from recurrent bacterial infections [10], which may suggest an impaired neutrophil function [11].

An increase of PMN-MDSC in peripheral blood has been reported to correlate with disease progression and promote resistance to therapy [12–14] in MM patients. Moreover, PMN-MDSCs have been found in the bone marrow, where they produce both ROS and arginase-1 in order to inhibit T-cells [13, 15] and to increase the proliferation of MM plasma cells *in vitro* [15].

ROS and arginase-1 have been reported to be main effectors of T-cell inhibition by PMN-MDSC and activated NDG [4, 7, 13, 15–17]. ROS inhibits T-cells through several mechanisms, including inhibition of DNA synthesis and by alteration of T-cell receptor signaling [18]. For example, ROS downregulates the CD3*ζ*-chain, making T-cells anergic to stimuli. Arginase-1 depletes L-arginine from the microenvironment, which also leads to downregulation of the CD3*ζ*-chain [19]. Romano et al. have shown that peripheral blood NDGs from MM patients inhibit T-cell proliferation through arginase-1, and that they have an altered gene expression profile compared to healthy NDG [7]. The role of bone marrow-derived NDG has not yet been investigated in MM. Since MM is a disease of the bone marrow and MM plasma cells are highly dependent on the bone marrow microenvironment for survival, it is important to unravel which role the NDG may play in the bone marrow. In this study, we investigated if blood and bone marrow NDG from MM patients have the ability to suppress T-cell responses, in the same way as MDSCs have been described to do. We found that NDGs become activated when they are in coculture with activated T-cells, and that blood NDGs from healthy individuals are as suppressive as MM NDG. Most interestingly, we found that bone marrow NDGs from MM patients suppress T-cell proliferation, while healthy bone marrow NDGs do not have this ability.

## 2. Material and Methods

### 2.1. Patients and Controls

Patients referred to the Department of Hematology, Skåne University Hospital, in Lund, Sweden, due to newly discovered M component or other symptoms consistent with plasma cell disorder were consecutively included during the period 2018 to 2020. These patients are referred to as newly diagnosed MM (NDMM, *n* = 16) and have not yet been treated for MM. A few patients with previously diagnosed MM (PDMM, *n* = 5) were also included in the study, and these patients have been treated for their disease. All patients were diagnosed with MM according to the International Myeloma Working Group Criteria. None of the patients had any ongoing symptoms of infection. Bone marrow and peripheral blood were collected from a total of 21 patients and 31 healthy controls.

The study was approved by an Independent Ethics Committee, The Regional Ethical Review Board in Lund, Sweden, Ref No. 2016/768. Samples were taken after the participants had signed a written informed consent.

### 2.2. Normal Density Neutrophil Isolation

NDGs were isolated from the RBC layer after a density gradient centrifugation using Lymphoprep (Stemcell Technologies, Cambridge, UK). The RBC pellet was lysed using 0.84% ammonium chloride (NH_4_Cl) for 15 min, and the NDGs were isolated using an EasySep™ Human Neutrophil Isolation Kit (Stemcell Technologies) according to the manufacturer's instructions. Blood NDGs with a purity of >95% were used for further analysis. The 1-5% that were not neutrophils did not form any clear populations. Most of it was debris, mixed with a few lymphocytes or monocytes. The bone marrow NDG had a purity of 90%, where the remaining 10% were mostly debris and a few lymphocytes.

### 2.3. T-Cell Proliferation Assay

Mononuclear cells were collected from the peripheral blood of healthy donors using Sodium Heparin Vacutainer™ CPT tubes (BD, Franklin Lakes, NJ, United States). T-cells were then isolated from the mononuclear cell layer using an EasySep™ human T-cell isolation kit (Stemcell Technologies) according to the manufacturer's instructions. The purity of the T-cells was >97%. The 1-3% that were not T-cells were mostly debris or an occasional monocyte. The purified T-cells were labeled with 1 *μ*M carboxyfluorescein succinimidyl ester (CFSE) (BD Horizon, Franklin Lakes, NJ, United States) in 1 ml PBS and washed. CFSE-labeled T-cells (10^5^ cells) were then added to a flat-bottom 96-well plate (Eppendorf, Hamburg, Germany) coated with anti-CD3 monoclonal antibodies (1 *μ*g/ml, clone OKT-3, Invitrogen, Carlsbad, CA, United States) and anti-CD28 monoclonal antibodies (2 *μ*g/ml, clone CD28.2, Invitrogen). The culture medium used was RPMI-1640 without L-glutamine (Sigma, Malmö, Sweden) supplemented with 10% fetal calf serum (Gibco™, Thermo Fisher, Waltham, MA, United States), 10^4^ U/ml penicillin (Gibco™, Thermo Fisher), 10 ng/ml streptomycin (Gibco™, Thermo Fisher), and 2 mM L-glutamine (Gibco™, Thermo Fisher). NDGs (50000 cells) were then added to the healthy T-cells (100000 cells) at a 1 : 2 ratio, yielding a final volume of 200 *μ*l/well. In some experiments, 1 : 1, 2 : 1, and 3 : 1 ratios were also tested. The effect of the ROS inhibitor catalase (Sigma, Malmö, Sweden) and the arginase inhibitor nor-NOHA (300 *μ*M, AH diagnostics, Solna, Stockholm) was also tested in some experiments. N-formylmethionyl-leucyl-phenylalanine (fMLF) was also used in some experiments, in order to activate the neutrophils. After 3 days of coculture, the supernatant was saved and stored at -80°C. The proliferation of the CFSE-labeled T-cells was measured on an Accuri C6+ flow cytometer (BD).

### 2.4. Cytokine Production Measurement

Interferon gamma (IFN-*γ*) production was measured in the supernatants using a Quantikine ELISA (R&D systems, Minneapolis, MN, United States) according to manufacturer's instructions. All samples were diluted 1 : 50.

### 2.5. Flow Cytometry

Whole blood and bone marrow samples were lysed using 0.84% NH_4_Cl and stained with the following antibodies: CD14—PerCP Cy5.5 (clone: M5E2, BD), CD80—FITC (Santa Cruz Biotechnology, Dallas, TX, United States), Siglec8—PE (clone: 7C9, BioLegend, San Diego, CA, United States), CD16—APC H7 (clone: 3G8, BD), CD66b—Alexa flour 700 (clone: G10F5, BioLegend), CD184—APC (clone: 12G5, eBioscience, San Diego, CA, United States), CD11b—BV785 (clone: ICRF44, BioLegend), CD62L—BV650 (clone: DREG-56, BioLegend), CD193—V510 (clone: 5 E8, BD), CD45—V450 (clone: 2D1, BD), HLA-DR—PE Cy7 (clone: L243, BD), and CD69—PE/DAZZLE (clone: FN50, BioLegend). The samples were then analyzed on FACS Aria Fusion (BD), for gating strategy see supplement figure 1. Single cells were gated, whereupon neutrophils were characterized as CD45^+^CD14^−^CD193^−^ cells and then further divided into mature, immature, and CD11b^−^ immature neutrophils based on their expression of CD11b and CD62L. When investigating the level of activation before and after isolation, as well as when analyzing the purity, the same antibody panel was used but the analysis was performed on Cytoflex (Beckman Coulter, Brea, CA, United States). All data was then analyzed using the Kaluza software (BD).

### 2.6. Statistical Analysis

Statistical analysis was performed using GraphPad Prism Version 8 (GraphPad Software, San Diego, CA, United States). The data is not normally distributed; therefore, nonparametric tests were used. Wilcoxon matched pairs signed rank test or Mann–Whitney was used to compare groups. Spearman correlation test was used to analyze the correlation between age and suppressive ability.

## 3. Results

### 3.1. Patient Characteristics

In this study, 16 untreated NDMM patients and 5 PDMM patients, who were referred to the Department of Hematology, Lund, Sweden, were enrolled. The clinical information of each patient can be viewed in supplementary table 1. There was no obvious difference between NDMM and PDMM NDG in any of the experiments, and they were therefore put in the same group, referred to as MM patients. Out of the 21 patients, 13 were male and 8 were female. The median age of the patients was 69 years of age, while it was 40 years of age for the healthy controls. Out of 31 controls, 11 were male and 20 were female.

### 3.2. Peripheral Blood Normal Density Granulocytes (NDGs) Inhibit T-Cell Proliferation

In order to investigate the suppressive effect of peripheral blood NDG on T-cell proliferation, NDGs were isolated and cocultured with healthy donor T-cells. After 3 days in coculture, T-cell proliferation and IFN-*γ* production were measured. IFN-*γ* is mainly produced by activated CD8^+^ T-cells as well as T-helper type 1 (Th1) cells, and IFN-*γ* reduction in the supernatant is commonly used to measure the suppressive activity of MDSC [1].

**Figure 1:**
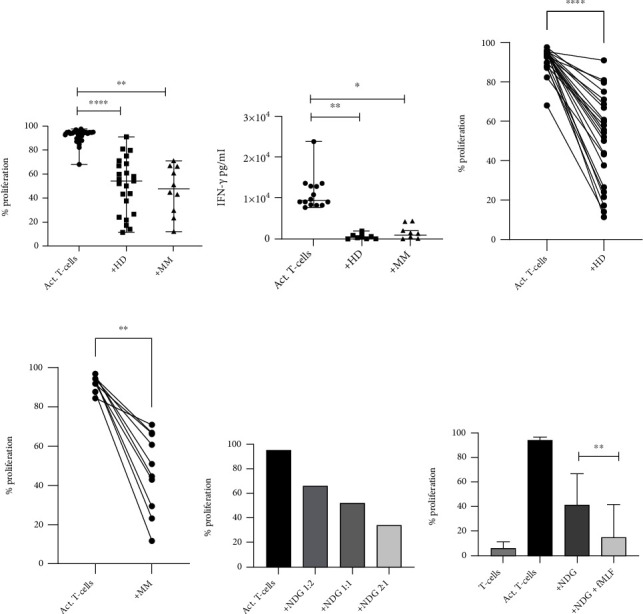
Inhibition of T-cell proliferation and IFN-*γ* production by peripheral blood normal density granulocytes. (a) Inhibition of proliferation with healthy donor (HD) and MM NDG. HD NDG inhibits T-cell proliferation (*p* < 0.0001), and the median proliferation is 54% (95% CI: 38–67). MM NDG also inhibits T-cell proliferation (*p* = 0.002), and the median proliferation is 48% (95% CI: 23–67). (b) The median of IFN-*γ* production for each group is 9400 pg/ml (95% CI: 820–13500), 460 pg/ml (95% CI: 0–1900), and 900 pg/ml (95% CI: 0–2000), respectively. Both HD NDG (*p* = 0.0078) and MM NDG (*p* = 0.0312) inhibit the production of IFN-*γ*. (c) The inhibitory effect of HD NDG (*p* < 0.0001) differs between 5 and 84% and (d) between 13 and 75% for MM NDG (*p* = 0.0156). (e) The inhibition of T-cell proliferation by NDG is dose dependent (data from representative experiment). (f) NDGs become more inhibitory in the presence of the activator fMLF (*n* = 9) (*p* = 0.0078). Error bars indicate median with range. Statistical significance was tested with Wilcoxon matched pairs signed rank test.

Healthy blood donor NDGs, which have not been preactivated with a neutrophil activator, inhibit T-cell proliferation (*p* < 0.0001, inhibition range 5–84%) as well as the production of IFN-*γ* (*p* = 0.0078). Peripheral blood NDG from MM patients inhibits both proliferation (*p* = 0.002 inhibition range 13–75%) and IFN-*γ* production (*p* = 0.0312) (Figures 1(a)–1(d)). Since there are age differences between the healthy donor group and the patient group, a correlation between age and suppressive ability was calculated. No correlation between the age and the suppressive ability of blood NDG was found, indicating that the suppressive ability of blood NDG is not dependent on age (data not shown). The inhibition by NDG was, however, dose dependent (Figure 1(e)). Since an inhibition was observed already at 1 : 2, this ratio was used for all experiments unless indicated. Mixing NDG from one donor with T-cells from another did not induce alloreactivity (supplementary figure 2). These data show that unstimulated peripheral blood NDGs have an inhibitory effect on T-cells.

Several groups have shown that blood NDGs become suppressive when they are activated. We therefore investigated if activation could potentiate the inhibitory activity of NDG. When peripheral blood NDGs were activated with fMLF, we saw an increased inhibition compared to nonactivated NDGs (*p* = 0.0078) (Figure 1(f)).

### 3.3. Efficient T-Cell Suppression by Bone Marrow Normal Density Granulocytes from Myeloma Patients

How different immune cells within the bone marrow are affected by the presence of malignant plasma cells is largely unknown. Therefore, we wanted to investigate if NDG from the malignant bone marrow had a different effect on T-cell proliferation than NDG from healthy donors. Bone marrow NDGs were isolated by magnetic separation and cocultured with T-cells. The isolated bone marrow NDGs contain cells from different stages of maturation.

**Figure 2:**
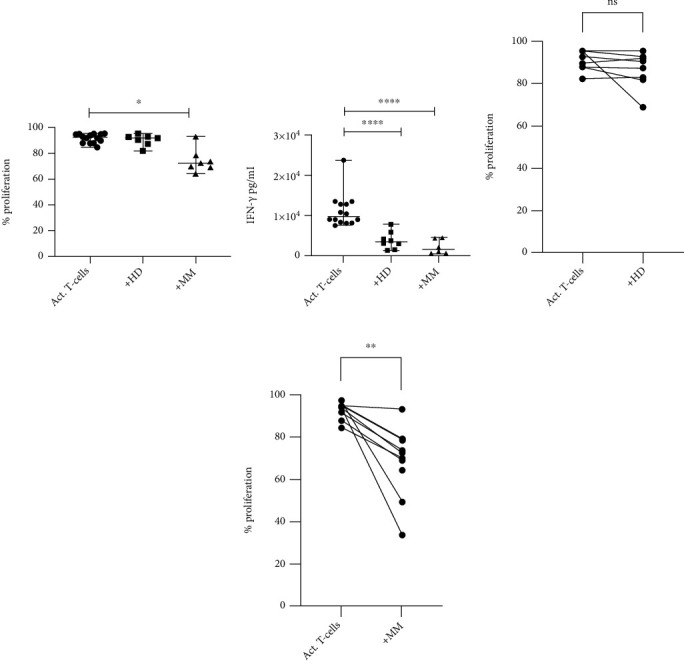
Inhibition of T-cell proliferation and IFN-*γ* production by bone marrow NDG. (a) Median proliferation for each group is 94% (95% CI: 88–95), 91% (95% CI: 82–93), and 71 (95% CI: 50–79), respectively. The proliferation of T-cells was not inhibited by HD NDG (*p* = 0.1016) but by NDG from MM patients (*p* = 0.002). (b) The median IFN-*γ* production for each group is 9700 pg/ml (95% CI: 8200–13500), 3500 pg/ml (95% CI: 1450–7800), and 1600 (95% CI: 600–4500), respectively. Both HD NDG (*p* = 0.0078) and MM NDG (*p* = 0.0312) have the ability to inhibit IFN-*γ* production. (c) The effect of HD NDG on T-cell proliferation (*p* = 0.1016) differed between +2% and -26% proliferation. (d) Inhibition by NDGs from MM patients (*p* = 0.002) differed between 1.7 and 60%. Error bars indicate median with range. Statistical significance was tested with Wilcoxon matched pairs signed rank test. Normal density neutrophils become activated by T-cells over time.

Bone marrow NDG from MM patients inhibited T-cell proliferation (*p* = 0.002, inhibition range 1.7–60%). Interestingly, NDG from healthy donors did not show an inhibitory effect (*p* = 0.1016, inhibition range +2–26%) (Figure 2). Since there are age differences between the healthy donor group and the patient group, a correlation between age and suppressive ability was calculated. No correlation between the age and the suppressive ability of bone marrow NDG was found, indicating that the suppressive ability of bone marrow NDG is not dependent on the age of the donor (data not shown). The IFN-*γ* production was inhibited by both MM NDG (*p* = 0.0312) and healthy donor NDG (*p* = 0.0078). These data indicate that bone marrow NDGs from MM patients suppress T-cell proliferation, while bone marrow NDG from healthy donors only showed a minor effect.

Next, we wanted to investigate if the suppressive effect observed in the blood was due to activation of NDG during the isolation process. Whole blood, as well as freshly isolated NDG from the same healthy donors, was stained with an antibody panel including the neutrophil activation markers CD11b and CD66b. No differences were observed (supplementary figure 3), indicating that the cells were not activated during the isolation process.

**Figure 3:**
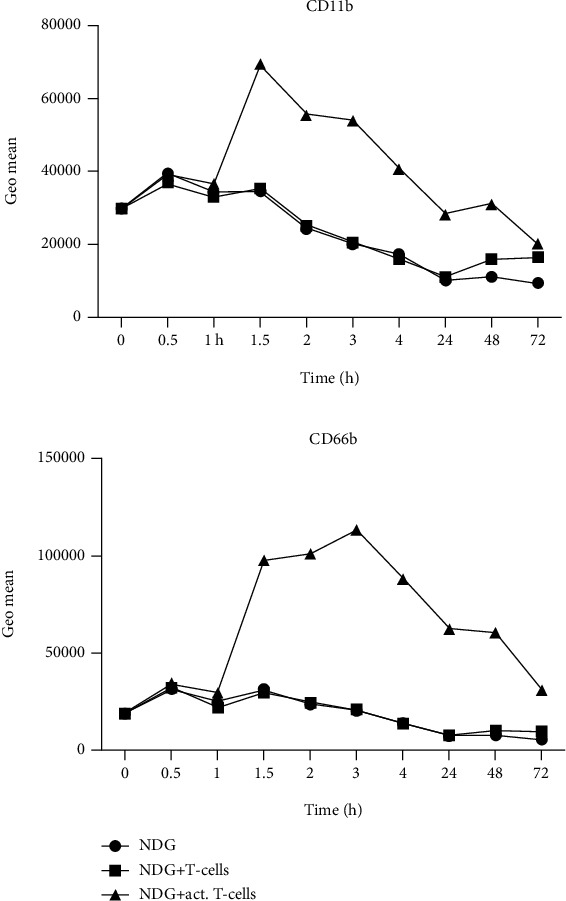
Neutrophil activation markers CD11b and CD66b increase during coculture with CD3/CD28 activated T-cells. (a) CD11b expression over time (72 h) on NDG alone in culture (ring), on NDG in coculture with CD3/CD28 activated T-cells (square), and on NDG in coculture with nonactivated T-cells (rectangle). (b) CD66b expression over time. These are representative plots from two experiments.

Since a T-cell inhibitory effect still can be observed, we hypothesized that NDGs are activated during the coculture, and that this late activation caused the inhibition. Hence, the expression of CD11b and CD66b on NDG was measured at several time points. After a few hours in culture, both in the presence of activated T-cells and alone, the number of viable NDGs starts to decline (data not shown). However, before the decline in viable NDG, we observed an increase in both CD11b and CD66b (Figure 3). The peak of CD11b was observed at an early stage, around 1.5–2 h after the addition of NDG to the culture. CD66b peaked at 2–3 h. The expression of both CD11b and CD66b slowly declined over time, as the viability of the cells went down. Culture of NDG alone did not show any signs of activation, nor did NDG cocultured with nonactivated T-cells. These data suggest that NDGs become activated during coculture with activated T-cells.

### 3.4. Catalase Can Restore the Proliferation

**Figure 4:**
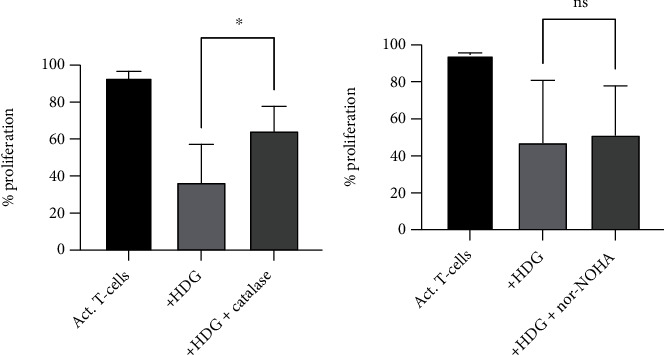
Catalase partially recovers the proliferation of T-cells. (a) Addition of the ROS inhibitor catalase to the coculture reverted the inhibitory effect of healthy blood NDG (*n* = 6), and T-cell proliferation was partially recovered (*p* = 0.0312). (b) When adding the arginase inhibitor nor-NOHA, the proliferation of T-cells was not recovered (*p* = 0.780) (*n* = 10).

Next, we wanted to test if the inhibitory effect of NDG was dependent on the production of ROS and/or arginase-1. Healthy T-cells were cultured in the presence of healthy donor NDG, either together with the ROS inhibitor catalase or the arginase inhibitor nor-NOHA. Catalase converts extracellular hydrogen peroxide (H_2_O_2_) into water and oxygen, while nor-NOHA blocks the active site of arginase-1. The presence of catalase and nor-NOHA alone did not have an effect on T-cell proliferation. Addition of catalase restored the proliferative capacity of T-cells (*p* = 0.0312) with 7–54 percentage units (Figure 4(a)), indicating that the inhibitory effect of blood NDG on T-cells is at least partly mediated by ROS.

The addition of nor-NOHA did not restore proliferation in the majority of the experiments (*p* = 0.780) (Figure 4(b)). In two out of ten experiments, the proliferation was restored with >20%, while nor-NOHA further inhibited or had no major effect on the proliferation in the other experiments. These data suggest that arginase-1 is not the main actor responsible for the T-cell inhibition induced by healthy donor NDG, while ROS play a more important role.

### 3.5. Levels of Immature and Mature Neutrophils in the Blood and Bone Marrow

**Figure 5:**
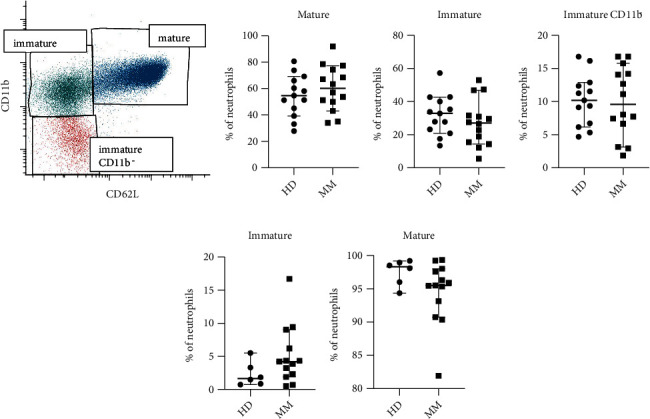
Percentage of mature, immature, and immature CD11b^−^ neutrophils in blood and bone marrow. (a) Gating strategy for the division of bone marrow neutrophils into mature, immature, and immature CD11b^−^ cells. The percentage of (b) mature, (c) immature, and (d) immature CD11b^−^ cells in the bone marrow of healthy donors (HDs) and MM patients. (e) The percentage of immature and (f) mature neutrophils in the blood of healthy donors and multiple myeloma patients. Statistical significance was tested with Mann–Whitney, but none was found.

Aarts et al. have shown that bone marrow-derived NDG with a mature phenotype have a superior inhibitory capacity compared to those with an immature phenotype [5]. We therefore wanted to investigate if the observed inhibitory effect when using bone marrow NDG from MM patients, not healthy controls, could be dependent on an increased frequency of mature NDG. To test this, both bone marrow and whole blood were evaluated with a granulocyte antibody panel and analyzed with flow cytometry. Gating strategy can be viewed in supplementary figure 1. The neutrophils were divided into mature, immature, and CD11b^−^ immature cells based on their expression of CD11b and CD62L (Figure 5(a)). The CD11b^−^ immature cells are more immature than the cells that do not lack CD11b expression. In the bone marrow, there was no significant difference in the frequency of mature, immature, and immature CD11b^−^ cells between healthy donors and patients (Figures 5(b)–5(d)). These data indicate that the increased inhibitory effect of bone marrow NDG from MM patients is not due to a skewed ratio of immature and mature neutrophils in MM patients. In the blood (Figures 5(e) and 5(f)), there was a tendency towards an increase of immature cells in MM patients.

## 4. Discussion

In the past decade, a regulatory function of neutrophils has been observed in both health and disease. LDG and PMN-MDSC were the first neutrophil subsets to be described as immune regulatory, and recently, a similar role has been demonstrated for activated NDG. In this study, we show that bone marrow NDGs from MM patients have the ability to suppress T-cell proliferation, while bone marrow NDGs from healthy individuals do not. We also show that NDGs do not have to be preactivated to have an inhibitory effect on T-cells. Instead, we show that they became activated over time during the coculture with activated T-cells.

Previous reports have shown that peripheral blood NDGs, which have been activated by a neutrophil activator, can exhibit MDSC function, e.g., suppress T-cell proliferation [4–8]. However, our data indicate a difference from these previous publications, since we observed an inhibitory effect with NDG, in the absence of neutrophil activators, when using a similar method. In line with our observation, de Kleijn et al. saw a minor inhibitory capacity of nonactivated blood neutrophils when cocultured with PHA-activated CD3^+^ lymphocytes [20].

There are several different methods that can be used for neutrophil isolation that all potentially can affect the function of the cells. In this study, we see no evidence of NDG activation by the isolation process, in terms of surface expression of CD11b and CD66b. Instead, our data suggest that NDGs become activated by coculture with activated T-cells. No activation was observed when cocultured with nonactivated T-cells, indicating that T-cell activation might be an important factor for neutrophil activation and requisite for neutrophil-mediated suppression.

An interesting finding in this study was that bone marrow NDG from healthy donors did not have an effect on the proliferation, but only on the IFN-*γ* production. Bone marrow NDG from MM patients had the ability to inhibit both. The reason for this partial inhibition when using bone marrow NDG from healthy donors remains unknown. In the MDSC literature, the absence of IFN-*γ* in the supernatant after coculture with T-cells is a sign of inhibition [1]. IFN-*γ* is largely produced by activated CD8^+^ T-cells as well as activated CD4^+^ Th1 cells, and our findings may suggest an inhibition of these particular subsets while other subsets still have the ability to proliferate in the presence of healthy donor bone marrow NDG.

ROS have been suggested to inhibit T-cells through several mechanisms, including inhibition of DNA synthesis and alterations in T-cell receptor signaling [18]. In this study, we observed that the inhibitory effect of NDG can be partially restored by the addition of catalase, suggesting that NDGs inhibit the T-cell proliferation partially through ROS production. This is in line with data from other groups, who have looked at NDG from healthy donors [5, 8, 21, 22].

A limitation in our study is that the age of the control group differs from the age of the patient group, where the age of the healthy donors is lower. It is well known that the function of neutrophils changes with age, usually providing a poorer function in the elderly [23]. For example, neutrophils from elderly people have a reduced ability to produce ROS when stimulated, as well as a reduced ability to perform phagocytosis [23, 24]. However, the spontaneous release of ROS seems to be higher in elderly people than young adults [25]. As the function of neutrophils from elderly is supposed to be less potent, and the ROS production after stimulation is decreased, we should observe a decreased inhibitory effect when using neutrophils from older patients. In this study, we do, however, observe a similar inhibitory effect in the blood as well as an increased inhibitory effect in the bone marrow suggesting that the inhibitory effect observed in the bone marrow is not dependent on the age of the donor but more likely the disease of the patient.

An earlier report has shown that NDGs from the peripheral blood of MM patients are chronically activated and inhibit PHA-induced T-cell proliferation [7]. These findings are not supported in this study as we do not observe an increased inhibitory effect of MM NDG isolated from the blood compared to NDG from healthy donors. We only observed an increased inhibitory effect of NDG from MM patients when using bone marrow NDG.

In systemic lupus erythematosus, NDGs have been suggested to restrict the proliferation of T-cells in an arginase-dependent manner [26]. The same thing has been suggested for blood NDG from both chronic myeloid leukemia patients [17] and MM patients [7]. Our data do not support an arginase-dependent inhibition. However, it is possible that both ROS and arginase-1 play an important role, but that it differs in disease phenotypes and experimental settings. Germann et al. have seen that peripheral blood NDGs from patients with colon cancer inhibit T-cell proliferation, and that the inhibitory effect can be rescued through the addition of a transforming growth factor beta (TGF-*β*) inhibitor. They suggest that NDG-secreted matrix metalloprotease MMP9 activates TGF-*β* in the microenvironment and thereby promote the inhibition of T-cells [27]. Moreover, there are studies suggesting that the inhibitory effect is either contact dependent [20, 28] or mediated through soluble factors [26]. The integrin Mac-1 (CD11b/CD18) has also been suggested as important for the inhibitory effect of NDG [4, 28]. When CD11b is blocked on activated NDG, they are prevented from exhibiting MDSC function [4]. However, another study has claimed that CD11b only plays a minor role, and that the upregulation of programmed death-ligand 1 (PD-L1) is more important for the inhibitory effect of NDG [20]. More studies are needed, where several inhibitory factors are investigated, in order to unravel how NDG inhibits T-cell responses in different diseases.

Bone marrow NDG from MM patients showed an increased T-cell suppressive effect compared to bone marrow NDG from healthy donors. It is well known that tumors can affect surrounding cells and promote a protumor environment, and it is possible that the tumor environment in the bone marrow preactivates the neutrophils and makes them more prone to inhibit T-cell proliferation. For example, the presence of TGF-*β* at a tumor site can mediate an “immunogenic switch” from proinflammatory antitumor N1 neutrophils, into anti-inflammatory protumor N2 neutrophils [29]. The presence of IFN-*γ* has also been suggested to promote a T-cell suppressive phenotype in NDG [20]. In MM, bone marrow PMN-MDSC and NDG produce soluble factors that protect human MM cell lines from the cytotoxicity of the MM treatments doxorubicin and melphalan [30].

The observed inhibitory effect of bone marrow NDG could be important for the growth of malignant plasma cells in MM patients. By inhibiting the T-cells, MM plasma cells get a greater chance to survive. This hypothesis is supported by the finding that T-cells from the bone marrow of MM patients display features of both exhaustion and senescence [31].

The frequency of mature, immature, and CD11b^−^ immature NDG did not differ between healthy controls and MM patients in our study. The observed difference in inhibition in the bone marrow can therefore not be explained by the frequency of mature and immature cells. Aarts et al. have reported that only mature bone marrow NDGs exert MDSC abilities when activated with fMLF [5]. If the maturity grade is important for the suppressive ability of bone marrow NDG in MM patients remains to be investigated.

It is evident that neutrophils from MM patients have an altered function, in both blood and bone marrow. In this article, we provide new information, by showing that NDGs isolated from the bone marrow of MM patients are more immune inhibitory than bone marrow NDGs from healthy donors. A deeper knowledge regarding the neutrophil-induced T-cell suppression could form the basis for a new therapeutic target in MM as bi-specific T-cell engagers and chimeric-antigen-receptor T-cells are new emerging therapies in MM.

## Data Availability

Data is not accessible due to ethical reasons (patient privacy).

## Supplementary Materials

Supplementary Materials Supplementary table 1: patient characteristics. Supplementary figure 1: gating strategy for whole bone marrow. Single cells were gated (not shown), whereupon the granulocytes were gated based on SSC-A and CD45 expression. Neutrophils were gated from the granulocytes based on CD193 and CD14 expression and then further divided into mature neutrophils, immature neutrophils, and more immature CD11b^−^ neutrophils based on CD11b and CD62L expression. Supplementary figure 2: the effect of allogenic and autologous cocultures on T-cell proliferation. (A) Allogenic coculture and (B) autologous cocultures effected the proliferation in a similar way, indicating that the suppressive effect is not due to alloreactivity. Supplementary figure 3: CD11b and CD66b on neutrophils before and after isolation with EasySep. (A) CD11b and (B) CD66b expression on whole blood neutrophils and neutrophils isolated with Stemcell Technologies EasySep Human Neutrophil Isolation Kit. CD11b and CD66b are neutrophil activation markers.
