# Supplementary material for: Bone Marrow Neutrophils of Multiple Myeloma Patients Exhibit Myeloid-Derived Suppressor Cell Activity
Source: J Immunol Res. 2021 Aug 6;2021:6344344. doi: 10.1155/2021/6344344 (PMC8369183; doi:10.1155/2021/6344344)
Supplement: Supplementary Materials — Supplementary table 1: patient characteristics. Supplementary figure 1: gating strategy for whole bone marrow. Single cells were gated (not shown), whereupon the granulocytes were gated based on SSC-A and CD45 expression. Neutrophils were gated from the granulocytes based on CD193 and CD14 expression and then further divided into mature neutrophils, immature neutrophils, and more immature CD11b− neutrophils based on CD11b and CD62L expression. Supplementary figure 2: the effect of allogenic and autologous cocultures on T-cell proliferation. (A) Allogenic coculture and (B) autologous cocultures effected the proliferation in a similar way, indicating that the suppressive effect is not due to alloreactivity. Supplementary figure 3: CD11b and CD66b on neutrophils before and after isolation with EasySep. (A) CD11b and (B) CD66b expression on whole blood neutrophils and neutrophils isolated with Stemcell Technologies EasySep Human Neutrophil Isolation Kit. CD11b and CD66b are neutrophil activation markers. [file 6344344.f1.docx]

Supplementary table I. Patient characteristics.

| **Pat No** | **Sex** | **Age** | **Diagnosis** | **ISS** | **rISS** | **FISH** | **M component class** | **M protein (g/L)** | **% PCs BM** |
| --- | --- | --- | --- | --- | --- | --- | --- | --- | --- |
| 1 | M | 62 | PDMM | III | II | Dup(1q) | IgG kappa | 5 | 20-30 |
| 2 | M | 82 | PDMM | I | - | +1q and  Del(13q14) | IgA kappa | 4 | 3 |
| 3 | M | 77 | NDMM | I | II | Dup(1q),  t(14;16) and del(17) | IgG kappa | 3 | 13 |
| 4 | F | 77 | NDMM | II | II | None | IgG lambda | 41 | 23 |
| 5 | M | 63 | NDMM | II | I | None | IgG kappa | 15 | 18 |
| 6 | M | 80 | PDMM | II | II | High risk cytogenetics | IgG kappa | 22 | 3 |
| 7 | M | 46 | NDMM | I | II | Del(17p) | IgG lambda | 25 | 21 |
| 8 | M | 76 | NDMM | III | II | None | IgG lambda | 19 | 15 |
| 9 | M | 71 | PDMM | ND | ND | ND | IgA lambda | 20 | 53 |
| 10 | F | 84 | NDMM | II | ND | ND | IgG lambda | 30 | 13 |
| 11 | M | 81 | NDMM | II | - | N/A | IgA kappa + IgM kappa | 18 | 4 |
| 12 | F | 72 | NDMM | II | II | None | IgD lambda + lambda | 21 | 35 |
| 13 | M | 63 | NDMM | I | I | None | IgA lambda | 19 | 23 |
| 14 | M | 70 | NDMM | I | ND | ND | IgG | 6 | 0 |
| 15 | F | 64 | NDMM | II |  | None | IgG kappa | 45 | 16 |
| 16 | M | 67 | NDMM | I | I | None | IgG kappa | 42 | 21 |
| 17 | F | 68 | PDMM | II | I | None | BJ myelom lambda | - | 23 |
| 18 |  | 45 | NDMM | I | II | Dup(1q) | IgA lambda | 20 | 12 |
| 19 | M | 74 | NDMM | II |  | Dup(1q) | IgA lambda | 12 | 24 |
| 20 | F | 69 | NDMM | ND | ND | Dup(1q) | IgG kappa | 40 | 11 |
| 21 | F | 51 | NDMM | I | I | Dup (1q) | BJ kappa | 0 | 13 |

PDMM = previously diagnosed multiple myeloma

NDMM = newly diagnosed multiple myeloma

ISS= International Staging System staging at diagnosis

rISS= revised ISS

FISH = Fluorescence in situ hybridization

ND= Not Done

N/A= not applicable

***Supplementary figure 1. Gating strategy for whole bone marrow****. Single cells were gated (not shown), whereupon the granulocytes were gated based on SSC-A and CD45 expression. Neutrophils were gated from the granulocytes based on CD193 and CD14 expression, and then further divided into mature neutrophils, immature neutrophils and more immature CD11b^-^ neutrophils based on CD11b and CD62L expression.*

***Supplementary figure 2. The effect of allogenic and autologous co-cultures on T-cell proliferation.*** A) Allogenic co-culture and B) autologous co-cultures effected the proliferation in a similar way. Indicating that the suppressive effect is not due to alloreactivity.

***Supplementary figure 3. CD11b and CD66b on neutrophils before, and after isolation with EasySep.*** *A) CD11b and B) CD66b expression on whole blood neutrophils and neutrophils isolated with StemCell technologies EasySep Human Neutrophil isolation kit. CD11b and CD66b are neutrophil activation markers.*
